# Genetic Diversity and Population Structure Analysis of European Hexaploid Bread Wheat (*Triticum aestivum L.*) Varieties

**DOI:** 10.1371/journal.pone.0094000

**Published:** 2014-04-09

**Authors:** Nanna Hellum Nielsen, Gunter Backes, Jens Stougaard, Stig Uggerhøj Andersen, Ahmed Jahoor

**Affiliations:** 1 Nordic Seed A/S, Odder, Denmark; 2 Department of Molecular Biology and Genetics, Aarhus University, Aarhus, Denmark; 3 Department of Agricultural Sciences, Faculty of Life Sciences, The University of Copenhagen, Copenhagen, Denmark; 4 Department of Molecular Biology and Genetics - Department of Organic Breeding and Agrobiodiversity, Faculty of Agriculture, Kassel, Witzenhausen, Germany; 5 Department of Plant Breeding, The Swedish University of Agricultural Sciences, Alnarp, Sweden; Agriculture and Agri-Food Canada, Canada

## Abstract

Progress in plant breeding is facilitated by accurate information about genetic structure and diversity. Here, Diversity Array Technology (DArT) was used to characterize a population of 94 bread wheat (*Triticum aestivum L.)* varieties of mainly European origin. In total, 1,849 of 7,000 tested markers were polymorphic and could be used for population structure analysis. Two major subgroups of wheat varieties, GrI and GrII, were identified using the program STRUCTURE, and confirmed by principal component analysis (PCA). These subgroups were largely separated according to origin; GrI comprised varieties from Southern and Eastern Europe, whereas GrII contained mostly modern varieties from Western and Northern Europe. A large proportion of the markers contributing most to the genetic separation of the subgroups were located on chromosome 2D near the Reduced height 8 (*Rht8*) locus, and PCR-based genotyping suggested that breeding for the *Rht8* allele had a major impact on subgroup separation. Consistently, analysis of linkage disequilibrium (LD) suggested that different selective pressures had acted on chromosome 2D in the two subgroups. Our data provides an overview of the allele composition of bread wheat varieties anchored to DArT markers, which will facilitate targeted combination of alleles following DArT-based QTL studies. In addition, the genetic diversity and distance data combined with specific *Rht8* genotypes can now be used by breeders to guide selection of crossing parents.

## Introduction

Hexaploid bread wheat (*Triticum aestivum L.*) is one of the most important cereal crops in the world, covering an area of 217 mill ha in 2010 [1]. It developed through two natural hybridizations of diploid wheat grass species. First, *Triticum urartu* (containing the A genome) and most probably *Aegilops speltoides* (containing the B genome) intercrossed [2], [3]. Second, about 6000 BC, the domesticated subspecies *Triticum dicoccum*, cultivated emmer containing the AB genomes, intercrossed with *Aegilops tauschii* (goat grass, containing the D genome), resulting in hexaploid wheat (containing all three genomes, ABD) [3], [4]. During the last 20 years, the increase in wheat yield in Europe has faced stagnation. Simultaneously, the demand for food is increasing due to the growing world population and the dietary changes in countries with rapidly growing economies [5], [6]. In order to meet these challenges, genetic improvement based on exploitation of genetic resources is required. Nevertheless, investigation of the wheat genome has faced difficulties due to the large genome size of bread wheat (∼17,000 Mb) and the high proportion (∼80%) of repetitive sequences [7], [8]. Therefore, adequate tools for the investigation of the bread wheat genome are essential.

Several types of molecular markers have been used for wheat genetic studies. Application of Restriction Fragment Length Polymorphism (RFLP), Randomly Amplified Polymorphism DNA (RADP), Simple Sequence Repeat (SSR) and Single Nucleotide Polymorphism (SNP) markers have provided effective genotyping [9]. One example is a study with 512 whole-genome microsatellite loci, representing a mean marker density of 5.1 cM [10]. Efficient population structure analysis requires markers to be well-distributed across the whole genome, and Diversity Array Technology (DArT) provides whole-genome fingerprints, generally with a high marker density [11]–[13]. DArT markers are bi-allelic dominant markers [14], hence the homozygous and heterozygous states cannot be distinguished. Knowledge about the germplasm of European bread wheat is essential to enrich genetic diversity in order to increase yield and improve other relevant traits such as disease resistance. Strong bottlenecks during domestication and intensive breeding of bread wheat have resulted in a genetically narrow germplasm [3], [15], [16]. Roussel et al. [16] demonstrated an increase in the genetic similarity of European varieties and a qualitative variation of allelic composition of European wheat lines over time. Furthermore, differences in allelic composition were found between different geographical regions in Europe. These differences could be caused by different breeding practices and requirements, one being that intensive selection pressure in wheat breeding began earlier in Northern and Western Europe [16]. Additionally, differences along chromosomes can be caused by the introduction of certain germplasm in specific geographical regions. One example is the 1B/1R translocation that provided new resistance sources for wheat, and has been widely used across Europe [17], [18]. Likewise, altered dwarfing genes differentiate the European wheat varieties, and dwarfing genes were crucial for the green revolution [19], [20]. For example, Worland et al. [19] found the dwarfing gene *Rht8* to be widespread in Southern Europe.

A number of studies highlight the importance of investigating the genetic structure of a population for exploitation of genetic diversity; thereby broadening the genetic base of modern cultivars via a purposeful selection of parents [21], [22]. Knowledge about genetic diversity and population structure is key to further improvements, and evaluation of diversity in germplasm is essential for the effective use of genetic resources in breeding programs. Reasons for the presence of subgroups within larger germplasm populations can include differences in geographical origin, human or environmentally driven selection or genetic drift [23], [24]. To investigate the detailed genetic makeup of population subgroups, linkage disequilibrium (LD) analysis is an important tool. LD reflects the degree of linkage between loci referring to the nonrandom association of alleles at different loci. LD is affected by the number of effective recombinations between loci (when measured as D’ or r^2^) and by the number of the mutations at those loci (only when measured as r^2^) in the history of the respective genotypes [23]. Recombinations between loci of similar allelic state have no effect on LD.

Hao et al. [10] characterized allelic diversity, LD, and population structure in a collection of 250 Chinese wheat genotypes. Their genetic structure analysis revealed two groups; one consisting of modern varieties, and one of landraces. They found that LD decayed less rapidly in modern varieties than in landraces and that the modern varieties showed a lower degree of allelic diversity, presumably because of the selection imposed by breeding. As the level of LD differs between and along chromosome arms [14], [22], [25] characterization of LD within subgroups could help to identify differences in selective pressures acting on specific genomic regions.

In this study, DArT markers were used for population structure analysis of a population mainly consisting of European commercial bread wheat varieties. We identified two main subgroups within our population, and their genetic diversity and LD patterns were analyzed on a genome-wide scale. Our study revealed marked variation in allele diversity as well as LD levels both within and between the two subgroups. Breeders can now take advantage of this information when choosing crossing parents for breeding, either by trying to maximize genetic diversity across whole genomes or by focusing on specific agronomic traits linked to DArT markers.

## Materials and Methods

### Plant Materials

A collection of seeds from 94 hexaploid wheat (*Triticum aestivum L.*) genotypes from 16 different countries was obtained (Table 1). Seeds were provided by GBIS (Gene bank Information System of the IPK Gatersleben), Biotec Saaten-Union, GK (Cereal Research Non-Profit Ltd.), Lantmännen SW Seed, Sejet Plant Breeding and Nordic Seed A/S Plant Breeding (See accession numbers from Table 1). Since wheat is a self-pollinated crop, the varieties were assumed to be homozygous. The genotypes were selected to allow association studies of wheat tissue culture response. Therefore, the population includes varieties with tissue culture responses ranging from poor to excellent. Since tissue culture responses were only known for a few genotypes, a number of randomly selected genotypes were also included, prioritizing European winter wheat varieties (Table 1).

**Table 1:** Origin (Ori) of the 94 genotypes, Belgium (BE), Canada (CA), Germany (DE), China (CH), Denmark (DK), France (FR), England (UK), Hungary (HU), Italy (IT), Luxembourg (LUX), Netherlands (NL), Poland (PO), Ukraine (UKR), Sweden (SE), USA (US), Bulgaria (BU).

ID	Genotype	Ori	Year	ID	Genotype	Ori	Year	ID	Genotype	Ori	Year
1	Aida(TRI9654)	BE		33	Mariboss	DK	2006	65	CY-45^A^	HU	
2	Svilena	BU		34	NOS 895889	DK	2006	66	Dioszegi 200(TRI1199)	HU	
3	Chris W(TRI 9556)	CA		35	Jensen	DK	2007	67	GK Mini Mano	HU	
4	Jao-Czin(TRI8089)	CH		36	Skagen	DK	2008	68	Arpadhalom(TRI1231)	HU	
5	Kranich(TRI9680)	DE	1969	37	Naksskov	DK	2009	69	Beke(TRI7718)	HU	
6	Pericles*	DE	1992	38	Gedser	DK	2011	70	Martonvásári12(TRI16024)(MV 12)	HU	1982
7	Lindos*	DE	1994	39	Genius	DK	2011	71	Martonvásári15(MV 15)	HU	1985
8	Flair*	DE	1995	40	Xantippe	DK	2011	72	Martonvásári17(MV 17)	HU	1987
9	Tommi*	DE	2002	41	Amundsen	DK	2012	73	Florio(TRI4773)	IT	
10	Paroli*	DE	2004	42	Opus	FR	2001	74	Livorno(TRI 1201)	IT	
11	JB Asano*	DE	2005	43	Expert	FR	2001	NG	Pavone(TRI3856)	IT	
12	Tabasco*	DE	2006	44	Florett	FR	2003	75	Sagnitzer(TRI1114)	LUX	
13	Lucius*	DE	2006	45	Global	FR	2008	76	Bristol	NL	1999
14	Glasgow*	DE	2007	46	Kepler	FR	2012	77	Tulsa	NL	2000
15	Mulan*	DE	2007	47	Recital	FR		78	Lovink(TRI7918)	NL	
16	Kredo*	DE	2009	48	Versailles 24	FR		79	Hanka(TRI4802)	PO	1979
17	Ellvis*	DE	2011	49	Brigadier	UK	1995	80	Orbita	UKR	2008
18	Bombus*	DE	2012	50	Grommit	UK	1998	81	Sleipner	SE	1995
19	Foxtrott*	DE	2012	51	Robigus	UK	2002	82	Kosack	SE	1999
20	Hamlet(TRI11954)	DE		52	Xi-19	UK	2002	83	Stava	SE	1999
21	Encore*	DK	1995	53	Ochre	UK	2005	84	SW Gnejs	SE	1999
22	Pentium*	DK	1995	54	Alchemy	UK	2006	85	SW Agaton	SE	2000
23	Saraste*	DK	1995	55	Oakley	UK	2006	86	Certo	SE	2002
24	Solist*	DK	1998	56	Cordiale	UK	2006	87	Bravur	SE	2005
25	Wasmo(TRI29508)	DK	1998	57	Lear	UK	2009	88	Visir	SE	2005
26	Vip	DK	2000	58	Santiago	UK	2010	89	Loyal (SW)	SE	2006
27	Legron	DK	2001	59	Warrior	UK	2010	90	SW Harnesk	SE	2006
28	Abba	DK	2001	60	Denman	UK	2011	91	Vega^A^(TRI4379)	SE	
29	Ambition	DK	2003	61	GK Csonger	HU	1980	92	Lewis^A^(TRI9931)	US	
30	Gallicia	DK	2003	62	GK Kinesco	HU	1983	93	Chris^A^	US	
31	Hereford	DK	2005	63	GK Delibab	HU	1992				
32	Torkil	DK	2005	64	GK Elet	HU	1996				

Year refers to the first year in official trials. Empty cells: year unknown. ID refers to ID in PCA-plot (Figure 4). NG refers to no genotype data.

*Breeders varieties and lines have not been assigned accession number. Seeds are available from breeders on request. Some varieties were obtained from IPK gene bank (Gene bank Information System of the IPK Gatersleben) and accession-ID is given in brackets.

A Spring variety.

### DNA Extraction and Genotyping by Diversity Array Technology

Genomic DNA from the 94 different genotypes was extracted using the CTAB procedure (Cetyl Trimethyl Ammonium Bromide; [26]). Plant material was harvested at the seedling stage and freeze dried. After precipitation of the DNA with isopropanol, the DNA pellet was transferred into a new 1.5 ml reaction tube, and the DNA was washed two times with cold 75% ethanol and air dried. The final DNA was diluted with TE buffer (pH 8.0) to a concentration of 100 ng DNA per μl. The DNA was sent to Triticarte Pty Ltd (Canberra, Australia; http://www.triticarte.com.au/). Diversity array technology, version 3 wheat DArT array profiling, was performed with an array including 7,000 markers from wheat, durum and various *Triticum* sp (Results in Dataset S1). The marker quality was evaluated according to the individual marker related statistics as indicated by Triticarte Pty Ltd. The mean marker P-value, reproducibility and call-rate was 81, 100 and 97, respectively, thus indicating high overall marker quality. Genotyping of one accession, ‘Pavone’, was unsuccessful and thus the accession was excluded from the analysis. A total of 1,849 polymorphic markers were obtained from the version 3 wheat DArT array profiling of the 93 wheat genotypes. Only 1,435 of these polymorphic markers were mapped (data obtained from Triticarte Pty Ltd) and included in the dataset.

### Population Structure Analysis by Bayesian Clustering

**Figure 1:**
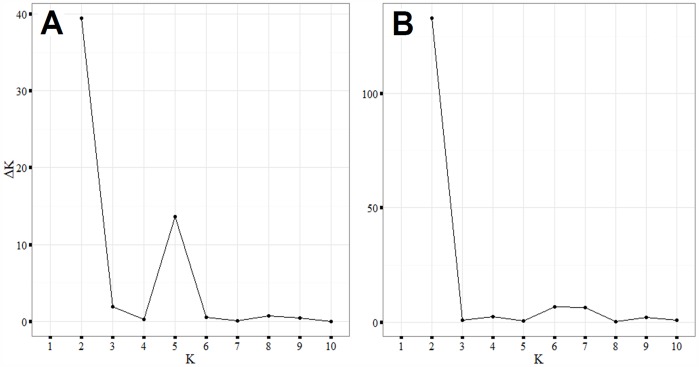
Estimation of the number of groups based on output from STRUCTURE-software. A: ΔK over K from 2–10 with the whole marker set of 1,849 markers B: ΔK over K from 2–10 with the reduced marker set of 695 markers.

The program STRUCTURE (version 2.1) was used to estimate the number of hypothetical subpopulations (K) and to estimate the membership probability of each genotype to the subpopulations [27]. A model-based (Bayesian clustering) clustering approach was performed, where the hypothesis of one to ten subpopulations was set and a Markov chain Monte Carlo (MCMC) of 9,999 burn-in phases followed by 9,999 iterations was run independently 10 times using an admixture model. The log-likelihood of the observed data Pr (X|K) for each value of K was obtained from the structure output (described in Pritchard et al. [28]). An *ad hoc* quantity analysis, based on the second order rate of change of the likelihood function, was performed (method described in Evanno et al. [29]) (Figure 1A). In the Bayesian clustering approach, Delta K peaked at a K-value (population number) of two. Thus, the population could be separated into two subgroups. Since the algorithms in STRUCTURE assume independent loci measured on randomly sampled unrelated individuals, another Bayesian clustering was done with a reduced marker set, where tightly linked loci were reduced to single haplotypes. The marker set was reduced by excluding markers with significant LD to markers already present in the set. The reduced marker set consisted of 695 markers and was used for structure analysis, AMOVA and gene diversity statistics. Also in the model-based (Bayesian clustering) clustering approach with the reduced marker set, two groups were indicated (Figure 1B). A possible separation into seven groups was also indicated; with the two major groups were divided into three and four groups, respectively. Since only 2% more of the total molecular variance was explained (using analysis of molecular variance (AMOVA) by seven compared to two groups, further analysis was based on two groups. The AMOVA was calculated between and within groups using GenAlEx v. 6.5 [30]. Next, the kinship-matrix of Jaccard’s distances [31] for the 93 genotypes was calculated based on the DArT markers using a Microsoft (Redmond, USA) Excel package programmed at the University of Copenhagen. Jaccard’s dissimilarity index was calculated as follows:
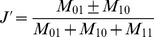



M_01_ represents the number of markers where accession *i* has no band (0) and accession *j* has a band (1); M_10_ represents the total number of markers where accession *i* has a band (1) and accession *j* has no band (0). M_11_ represents the total number of markers where both *i* and *j* have a band (double presence of the same allele). In Jaccard’s distance the case where both *i* and *j* are (0) is ignored, because this allelic state cannot be differentiated from missing data due to the dominant nature of the DArT markers. Using the same Microsoft Excel package, a Principal Coordinate Analysis (PCoA) based on the kinship distance matrix was carried out to visually check for genetic structure and genetic outliers. The variety, ‘Paroli’, was identified as an outlier from the PCA-plot (results not shown). Since ‘Paroli’ is a variety developed in Germany, it was not expected to be an outlier. Therefore, it has been assumed that the DArT analysis of this line failed, and the genotype has been discarded from further analysis. Principal component analysis (PCA) was used to visualize the subpopulations found in STRUCTURE. All graphics were done using the program R version 2.15.2 (http://cran.r-project.org). To detect markers with high influence on the grouping in the PCA, 5% of the markers (35 markers) having the highest PCA loadings were used. The 35 detected markers were confirmed by performing a General Linear Model (GLM) association between markers and the subgroup divisions. The separation into two groups found in the population structure analysis was confirmed by these two independent analyses. Both analyses revealed the same markers to influence the separation, except for two markers which were found in the GLM and not in the PCA loading analysis.

### PCR Conditions and Primers

The 93 genotypes were evaluated with three different primer sets (Table 2). Two of the PCR products were resolved using standard agarose gel electrophoresis. The SSR marker with M13 tailing (6-FAM) was separated using capillary electrophoresis on an ABI3130 Genetic Analyzer (Applied Biosystems, Foster, CA,USA).

**Table 2:** Primer sets and PCR conditions.

Name	ForwardPrimer	Reverseprimer	PCR conditions
RIS	taatttctgcttgctccatgc	actggggtgcactggattag	94°C for 4 min; 94°C for 15 s; 60° for 45 s; 72°C for 30 s for 35 cycles
L34SPF/L34DINT13R2	gggagcattatttttttccatcatg	actttcctgaaaataatacaagca	94°C for 4 min; 94°C for 15 s; 59° for 45 s; 72°C for 30 s for 35 cycles
L34DINT9F/L34MINUSR	ttgatgaaaccagttttttttcta	tatgccatttaacataatcatgaa	
Xgwm261-2D*	TGTAAAACGACGGCCAGtctccctgtacgcctaaggc	ctcgcgctactagccattg	1 cycle: 94°C for 4 min; 18 cycles: 94°C 1 min, 64°C 30 sec,72 1 min; 20 cycles: 94°C 1 min, 55°C 1 min,72°C 1 min; 1 cycle: 72°C 5 min

*Including M13-tale. Run with FAM on ABI.

### Linkage Disequilibrium Analysis

LD between the DArT markers was estimated using the TASSEL 2.1 software. The analysis comprised the pairwise estimated squared allele-frequency correlations (r^2^) and the significance of each pair of loci. The r^2^-values were calculated with comparison-wise significance computed using 1000 permutations. Breseghello and Sorrells [25] suggested comparison of all marker-pairs in the linkage analysis, including both intra- (pairs on the same chromosomes) and inter- (pairs between chromosomes) chromosomal pairs. In our LD analysis, only intra-chromosomal comparisons were included. To estimate the LD decay, significant r^2^-values (with p*-*values <0.05) were plotted against genetic distance (cM) between the loci-pairs, and a second-degree smoothed loess curve was fitted using the program R (www.R-project.org). The interception of the loess curve and background LD was considered as an estimate of LD decay [25]. The critical r^2^-limit in our study was not based on the method described by Breseghello and Sorrells [25], but on the assumption that all marker-pairs with a distance above 50 cM were un-linked [32]. Since only the intra-chromosomal pairs were computed, the background LD was determined by the 95th percentile of unlinked r*^2^*-values, referring to a LD between marker-pairs greater than 50 cM apart (described in Zhou et al. [32]).

## Results

### The Density of Polymorphic DArT Markers Differs between the A, B and D Genomes

**Figure 2:**
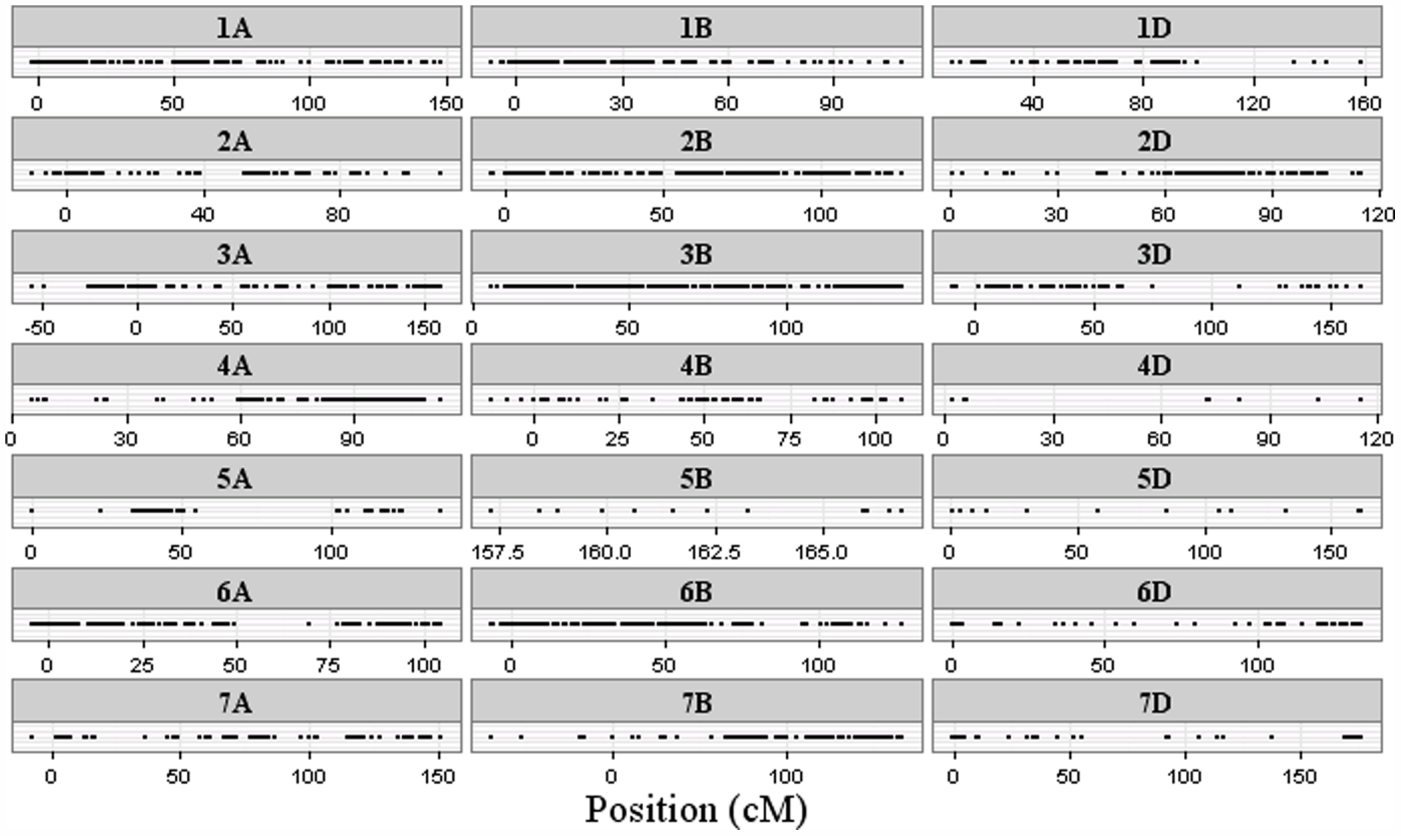
Chromosome coverage of 4,570 mapped DArT markers. Information provided by Triticarte Lty.

**Figure 3:**
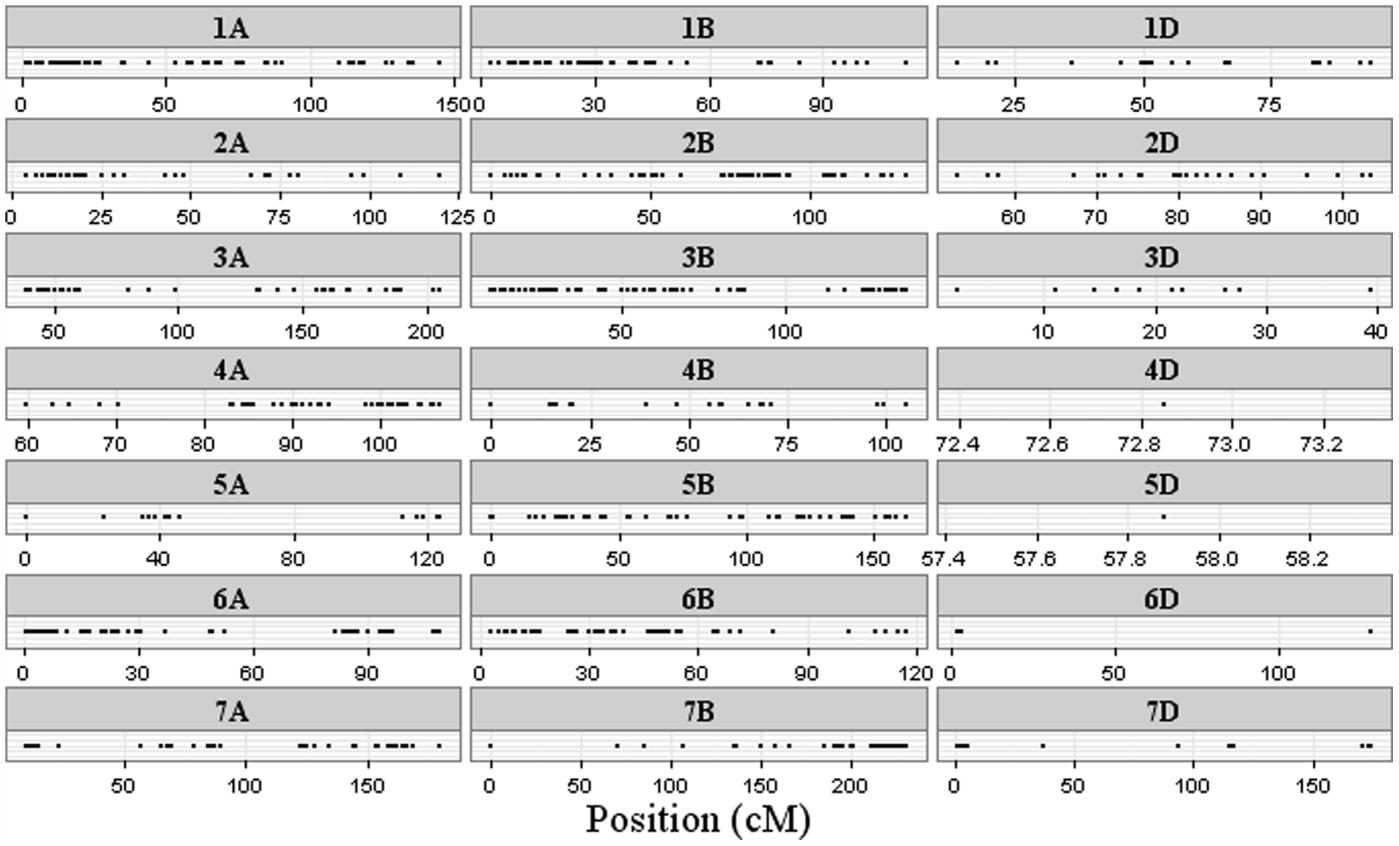
Chromosome coverage of polymorphic markers. Distribution of 1,435 polymorphic and mapped DArT markers. Information provided by Triticarte Lty.

The DArT marker array included 7,000 markers of which 4,570 were placed on the genetic map (Figure 2). Among the 4,570 informative markers, 48% were located on the B genome, 33% on the A genome and 19% on the D genome (Table S1). For all three genomes, more than 97% of the markers were mapped with a distance shorter than 10 cM, and neither the A nor B genome had gaps larger than 50 cM. However, the D genome had one large gap of 67 cM on the 4D chromosome. The B genome had one gap larger than 10 cM, while the A genome had 13 gaps larger than 10 cM. In summary, the largest and the highest number of gaps between DArT markers were found on the D genome. Of the 4,570 markers anchored on the genetic map, 1,435 were polymorphic (Figure 3). Most of these markers were located on the B genome, followed by the A and D genomes (Table S2). Polymorphism information content (PIC) values were computed by Triticate Pty Ltd and ranged between 0.16 and 0.38 with a mean value of 0.30. Nine markers were found to be unique (private) for single genotypes (Table S3). A total number of 150 alleles occurred in 5 or less of the lines, and most of these rare alleles were mapped to the A and B genomes. The chromosomes 1A, 6A, 2B and 3B contained the highest number of rare alleles.

### Separation into Subgroups by Structure Analysis was Correlated with Geographical Origin

**Figure 4:**
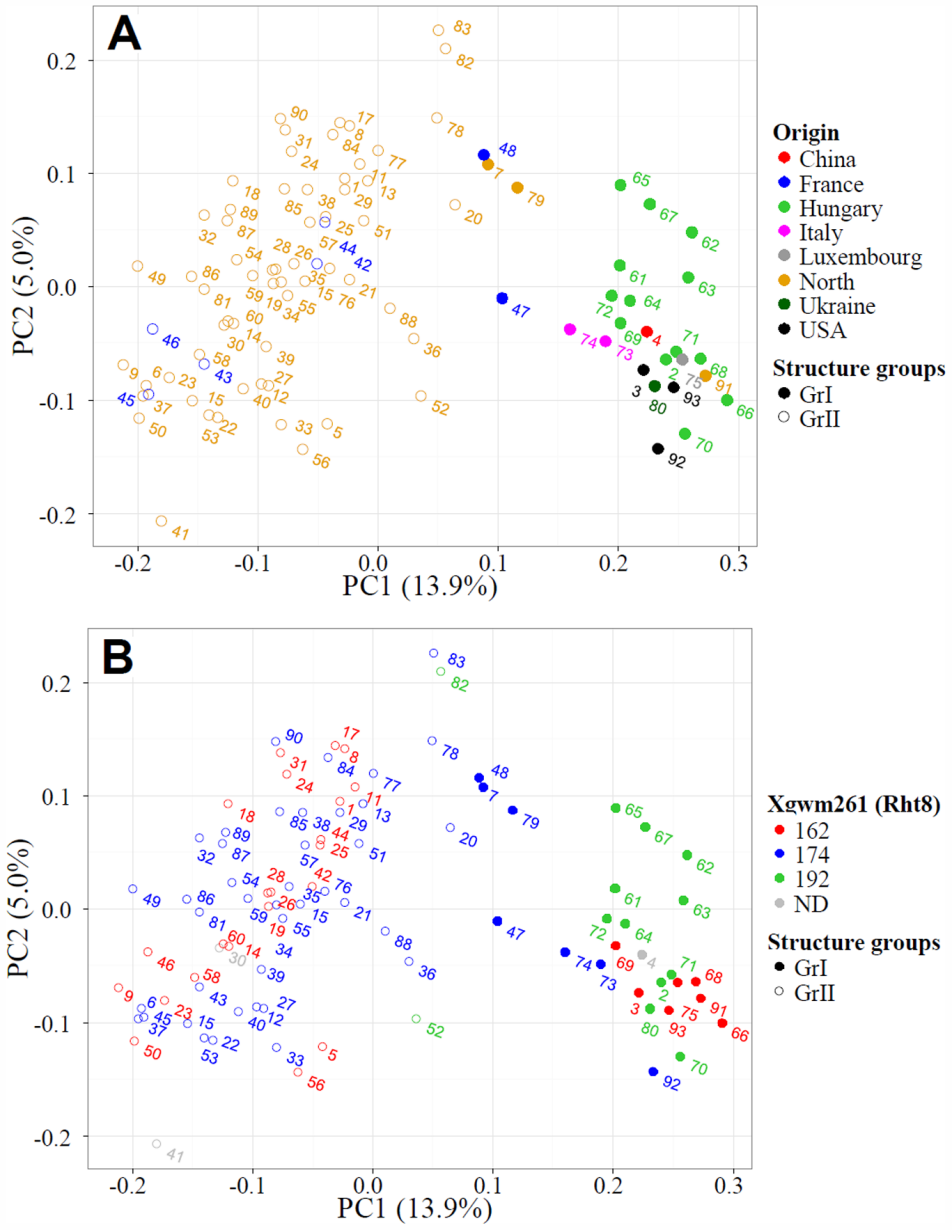
Principal component analysis of 92 hexaploid wheat genotypes. Based on analysis of 695 polymorphic DArT markers. Numbers refer to the genotypes described in Table 1. Closed circles: GrI. Open circles: GrII. **A:** Colored according to geographical origin. North: Sweden, Denmark, Germany and UK. **B:** Colored by the band size of the *Rht8* marker Xgwm261.

To analyze the genetic diversity within the population, the relatedness of the genotypes was investigated using population structure analysis. Based on the hypothesis of two subgroups, a Q-matrix was calculated using the output of the Bayesian clustering in Structure 2.1 [28]. The Q-values indicate the level of relatedness of each genotype to the two defined subgroups (Figure S1). Most of the genotypes from Hungary belonged to the smaller GrI subgroup, while varieties from Western and Northern Europe were associated with the second and largest subgroup (GrII). Varieties from Italy were part of GrI, while most varieties from France were part of GrII. Principal Component analysis (PCA) was used as an alternative way of visualizing the genotype data (Figure 4A). The first and second principal components explained 14% and 5% of the variation, respectively. Overall, GrI and GrII were clearly separated by the PCA. AMOVA showed a significant separation of the GrI and GrII (p-value 0.001), and 14% of the total variation was accounted for by differences between groups (Table S4). Additionally, the Fixation index (F_ST_) was calculated to measure the differentiation of the population in relation to genetic structure, and the results are shown in Table 3. Furthermore, the genetic diversity (H_p_) levels are indicated (Table 3) and were 0.384 for the entire population, while the fixation index was 0.127.

**Table 3:** Genetic diversity statistics for two subgroups: population size, no of private alleles, mean gene diversity (bold) within groups, pairwise F_st_ between groups.

Structure Group	Population size	No of private alleles	GrI	GrII
GrI	26	6	**0.384**	
GrII	66	53	0.127	**0.334**

### Linkage Disequilibrium Decayed Over 23 cM

**Figure 5:**
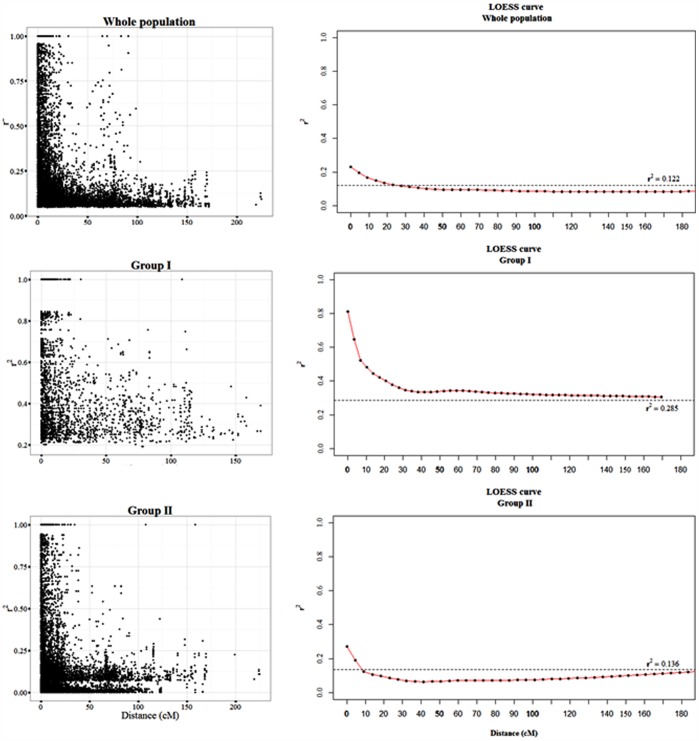
LD decay. Left panels show scatterplots of significant r^2^-values of intra-chromosomal marker-pairs as a function of genetic distance (cM). Right panels show the corresponding second-degree smoothed loess curves. The dashed line indicates the LD decay calculated using the 95th percentile of unlinked *r^2^*-values (marker-pair distance greater than 50 cM). r^2^ threshold values are shown on the plots.

LD was calculated for the entire population and for the two subgroups. The number of intra-chromosomal marker-pairs and the number of significant marker-pairs can be seen in Table 4. A total number of 43,549 intra-chromosomal pairs were detected in the population. Mean r^2^-values for the entire population and for the two subgroups were calculated for all 21 chromosomes (Table 5). The mean r^2^-value for the total population was found to be 0.080 for all marker-pairs and 0.271 for significant marker-pairs. The LD decay is illustrated in Figure 5 (right), including the second-degree smoothed loess curve. In order to determine the LD decay, the LD threshold was assessed by estimating a background LD (or critical r^2^-limit) for each subgroup and for the total population. The intercept of the loess curve was considered as the LD decay. The LD decay for the total population was found to be 23 cM. In GrI the LD decay did not intercept the background LD; however the loess curve extended to approximately 35 cM. GrII had a LD decay of 19 cM. Furthermore, the LD analysis revealed differences between the genomes, and the D genome had the highest r^2^-value, while the B genome showed the lowest (Table 5).

**Table 4:** Number of intra-chromosomal marker-pairs in the total population and in the two groups, respectively.

	Total pairs	No. significantpairs	% significantpairs	No. un-linkedpairs	% un-linkedpairs
Total population	43,549	11,393	26	17,005	39
GrI	35,889	3,527	10	14,736	41
GrII	42,846	10,552	25	16,891	39

Unlinked refers to a marker-pair distance >50 cM.

**Table 5:** Mean r^2^-value for the significant and non-significant intra-chromosomal marker-pairs in the total population plus the two groups (GrI and GrII) found in the population structure analysis.

Chromosome	GrI	GrII	Total pop
1A	0.108	0.069	0.067
2A	0.111	0.173	0.158
3A	0.111	0.106	0.094
4A	0.107	0.105	0.096
5A	0.135	0.093	0.09
6A	0.088	0.078	0.069
7A	0.086	0.055	0.055
**A**	**0.098**	**0.071**	**0.079**
1B	0.12	0.094	0.092
2B	0.105	0.074	0.067
3B	0.086	0.052	0.045
4B	0.131	0.134	0.14
5B	0.08	0.047	0.05
6B	0.073	0.051	0.043
7B	0.12	0.062	0.059
**B**	**0.09**	**0.064**	**0.06**
1D	0.17	0.486	0.155
2D	0.283	0.109	0.241
3D	0.395	0.545	0.463
4D	0.003	0.691	0.392
5D	–	–	–
6D	0.233	0.212	0.209
7D	0.042	0.178	0.154
**D**	**0.277**	**0.29**	**0.229**
**Whole genome**	**0.104**	**0.082**	**0.08**

The LD analysis showed a difference in the r^2^-values of the chromosome groups between the two groups (Table 6). Both structure groups had a higher average r^2^-value than the entire population (Table 5). GrI had the highest average r^2^-value and higher r^2^-values for the A and B genomes than GrII, while GrII had a higher r^2^-value for the D genome. To identify differences in LD between the total population and the two structure groups along the chromosome arms, the r^2^-values of all adjacent marker-pairs of all chromosomes were plotted on the genetic map (Figures S2–S4).

**Table 6:** Mean r^2^-value for the significant and non-significant intra-chromosomal marker-pairs of each chromosome group.

Chromosome group	GrI	GrII
1	0.13	0.22
2	0.17	0.12
3	0.2	0.23
4	0.08	0.31
5	0.11	0.07
6	0.13	0.11
7	0.08	0.1

The two groups (GrI and GrII) were found in the population structure analysis.

### Breeding for Rht8 Appears to have Contributed to Subgroup Separation

To investigate possible influences of breeding for agronomic traits on population structure, we examined the 35 markers that contributed the most to both subgroup and PCA separation. A number of these were located near markers for known traits. Specifically, 12 of the 35 markers were found on chromosome 2D near the *Rht8* locus (Table S5), suggesting that breeding for *Rht8* could have had a major impact on the genetic separation of the two subgroups. To verify this, the population was genotyped using a *Rht8* marker located on chromosome 2D (Xgwm261, [19]). The marker produced 162, 174 and 192 bp bands. Eleven of the thirteen varieties displaying the 192 bp band belonged to GrI, and 40 of the 47 varieties showing the 174 bp band belonged to GrII (Figure 4B and Table S6), supporting the idea that breeding for specific *Rht8* alleles has contributed significantly to the genetic structure observed within our population.

Two other markers for agronomic traits were tested using PCR genotyping. Firstly, the population was genotyped with a PCR marker for *Lr34* located on chromosome 7D (Table 2, [33]). *Lr34* alleles were present in the four varieties 80, 69, 72, and 92, all belonging to GrI. Secondly, the RIS-marker indicating the presence of any rye chromatin was scored [34]. This suggested a wheat-rye translocation in 14 lines (no. 2, 30, 32, 33, 37, 45, 55, 56, 57, 61, 70, 73, 81 and 86). Four of these lines (2, 61, 70 and 73) belonged to GrI and the rest to GrII (numbers in Table 1).

## Discussion

### The Distribution of DArT Markers Indicated that the D Genome is the Least Polymorphic

Marker-based population structure analysis requires well distributed and informative markers, which reflect the overall diversity of the genome. DArT markers have previously been found to cluster in particular regions [13], and in our study, gaps and marker clusters were also revealed. As an example, chromosome 1A, between 0 and 25 cM, showed a high density of markers (Figure 3). Nevertheless, the 4,570 mapped markers were distributed along all three wheat genomes. The number of markers was highest on the B genome and lowest on the D genome; thus, the largest marker-gaps were found on the D genome. The same distribution tendency was observed among the polymorphic markers. These observations are consistent with previously published results on the distribution of DArT markers between genomes [12], [13], [35]. Fewer polymorphic markers on the D genome indicate a lower frequency of effective recombinations due to a lower diversity of this genome [4], [15]. This was expected, since hexaploid wheat gathered a larger proportion of genetic diversity from its tetraploid ancestors than from *A. tauschii* (containing the D genome) during domestication, resulting in a higher number of effective recombinations in the A and B genomes relative to the D genome [15], [36]. Consequently, the highest average marker distances would also be expected on the D genome, which our study confirms (S2). The proportion of polymorphic markers is relatively low in wheat, (approximately 34% in [37]), and in our study only 26% of the DArT markers were polymorphic. The low fraction of polymorphic markers suggests a relatively narrow wheat gene pool in Europe.

Breeding for traits of agronomic importance such as yield, quality, and disease resistance has influenced allele-richness across the wheat genome [38], [39]. Four parameters can indicate specific allele-richness of chromosome parts: marker density, clustering of markers, and distribution of rare or polymorphic alleles. High marker densities were previously revealed on the 2A, 3B, 3D, 4A and 7A chromosomes [38], [40], and likewise clusters on the same chromosomes were seen in our study (Figure 2–3). Most rare alleles were found on the chromosomes 1A, 2B, 3B and 6A. Furthermore, the most polymorphic markers were found on 1A, 6A and 3B (data not shown). Near the location of rare alleles in our population, several disease resistance genes have been localized [41], [42]. As an example, a quantitative trait locus (QTL) in bread wheat for resistance towards Septoria tritici blotch (STB) was detected on the 3B chromosome [43]. Moreover, the leaf rust (*Puccinia triticina)* resistance gene *Lr10* has been mapped to chromosome 1A in bread wheat ([41]). On the 2B chromosome the genes *Yr32* and *Yr17* for yellow rust (*Puccinia sriiformis)* resistance have been detected [42].

### Long-range LD Likely Reflects Strong Breeding-driven Selection of European Varieties

For our population, LD decayed over 23 cM, whereas both Chen et al. [40] and Zhang et al. [22] found faster decays of 2.2 and 10 cM, respectively. The difference may be due to differences between populations and marker type and distribution. Chen et al. [40] investigated a Chinese wheat population including both modern varieties and landraces, while Zhang et al. [22] included both hard and soft winter wheat from a wide range of all regions of the United States. In contrast, our population represents a relatively limited geographical area. Furthermore, both studies used SSR markers [40], [44], while DArT markers were used in our study. In both GrI and GrII, the average r^2^-value was higher than for the total population (Table 5), indicating that more alleles are in LD in smaller population with limited geographical origin. Likewise, an extended LD decay was found in GrI compared to the total population (Figure 5). The higher LD of GrI may be attributed to the fact that GrI holds most of the Hungarian genotypes and within these blocks of alleles with high LD may be frequent. The LD decay of GrII (containing 69% of all lines) was similar to the LD decay of the total population. This corresponds to the findings of Neumann et al. [14], where LD decay of the largest group (containing 80% of all lines) was similar to the LD decay of the total population.

### Variations in LD Patterns Suggest that Different Selective Pressures have Acted on the Two Population Subgroups

**Figure 6:**
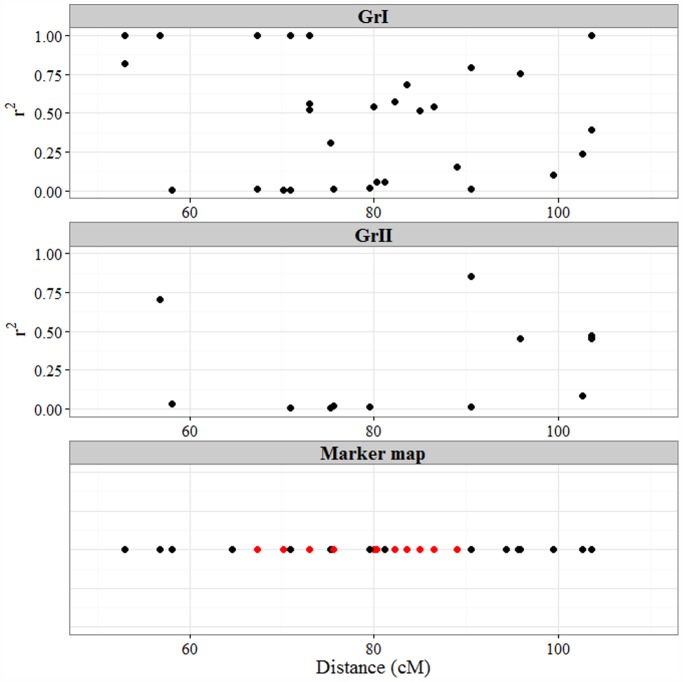
Linkage disequilibrium versus chromosome position on 2D. The two upper panels display r^2^-values for adjacent marker-pairs along chromosome 2D. The LD between pairs of adjacent loci is plotted at the locus nearest to zero. GrI and GrII indicate the two population subgroups. The lower panel shows the positions of individual markers on chromosome 2D. Markers with high contributions to subgroup separation are shown in red. See also table S5.

Previous results showed that LD differs between the chromosomes [10], [22], [25]. In line with the findings of Chen et al. [40] and Hao et al. [10], we found the highest levels of LD for markers located on the D genome (Table 5). It has been suggested that high LD levels can be a result of selection, which can increase the correlation among alleles at specific loci [45]. Hence, characterization of differences in LD levels between chromosomes can help to identify genomic regions, which have been subject to genetic selection. For example, relatively low r^2^-values between adjacent marker pairs on some chromosomal areas of 3A, 3B, and 2D (Figure 6 and Figures S3–S4) were found in GrII. In contrast, a greater number of high r^2^-value marker pairs were found in GrI on these chromosomes, indicating that varieties in the two subgroups could have been subject to different selective pressures in these genomic regions.

### Markers on Chromosome 2D Near the Rht8 Locus had a Major Impact on Population Structure

These candidate regions for differential selection could represent specific chromosomal areas that were targets in intensive breeding [16], [46], [47], and could also have an impact on the separation of population subgroups. In our study, the separation was controlled by a number of chromosome regions (Table S5). To identify agronomic traits that are linked to these chromosome areas, our results were compared to the study of Crossa et al. [46]. This comparison indicated the potential influence of selection based for a number of important agronomic traits (Table S5) including the 1B/1R locus, wheat dwarfing genes and day-length insensitivity and flowering time genes *(Ppd1* and *Vrn).* Strikingly, one third of the markers with largest contributions to the genetic separation of subgroups were located on chromosome 2D near the *Rht8* dwarfing locus (Table S5), and it is well known that dwarfing genes have been used to improve yield as one of the main strategies in modern European bread wheat breeding [48]. The strong impact on population structure of breeding for *Rht8* was validated using PCR-based markers, confirming that different *Rht8* alleles were found in GrI and GrII. The two PCR markers for *Lr34* and the wheat-rye translocation also showed some correlation with the subgroup separation, but did not distinguish as clearly between the groups as the *Rht8* marker. *Lr34* is linked to *Yr18* and these have provided durable resistance to leaf rust (caused by *Puccinia triticina*) and stripe rust (*Puccinia striiformis*), respectively. Moreover, the same chromosomal area has been associated with powdery mildew resistance against *Blumeria graminis* (DC) EO Speer f. sp. *Tritici* [49]. *Lr34* is rare in western European wheat, and it has been used extensively in spring wheat grown in the United States [49]. This corresponds well to our results, since we detected *Lr34* in a spring wheat variety from the United States (no. 93, Table 1), one from Ukraine (no. 80) and in two Hungarian lines (no 69 and 72). Several rye chromatins have been introduced into wheat lines to increase resistance to pests and pathogens [18], and are now present in some modern wheat varieties. Our results indicated a wheat-rye translocation in the variety Sleipner (No. 81, Table 1), where it has also previously been detected [50].

The microsatellite marker Xgwm261 has been widely used to detect the dwarfing gene *Rht8* [19], [51]–[53], and three major alleles generating products of 165, 174 or 192 bp have been described [53], [54]. These band sizes correspond well with band sizes found in our study (Table S6), except for the 162 bp band, which might be equivalent to the 165 bp band described in other studies [54]. Compared to the 174 bp variant, 162 bp alleles increase and 192 bp alleles reduce plant height [54]. The 192 bp band is associated with *Rht8* dwarfing alleles, which are widespread in varieties from South-East Europe. This corresponds well with our results where we found that the majority of lines with a band size of 192 originated from Hungary and belonged to GrI (Figure 4B). Most of the varieties with 174 bp alleles originated from Western Europe and belonged in GrII, which is in line with previous results describing 174 bp alleles in varieties from Western and Central Europe [54]. Hence, *Rht8* might not be responsible for reduced plant height in most Western and Northern European bread wheat varieties. *Rht8* is mapped to chromosome 2D, which was identified in our LD analysis as a candidate region for differential selection between the two population subgroups, as mentioned above. This is reflected by higher LD between adjacent marker pairs on chromosome 2D for GrI, where most varieties contain the *Rht8* allele causing reduced plant height, than for GrII (Figure 6).

### Population Structure and Genotype Data can Facilitate Selection of Crossing Parents

Knowledge about population structure and underlying selection for specific traits can assist the selection of crossing parents in order to combine diverse germplasm in a breeding program. The data presented here can be exploited in several ways. Crossing parents can be selected based on their genetic distance, simply to maximize overall genetic diversity and potential for genetic gain in the progeny. It also provides an overview of the allele composition of bread wheat varieties anchored to DArT markers, which will facilitate targeted combination of alleles following DArT-based QTL studies. Finally, the identification of the *Rht8* locus as a major contributing factor to the separation between the two genetic subgroups suggests the possibility of introducing *Rht8* dwarfing alleles into Northern and Western European bread wheat varieties. For this purpose, the varieties Xi-19 (Table 1 no. 52, UK) and Kosack (Table 1 no. 82, Sweden) may be of particular interest. They are the only GrII varieties with the 192 bp *Rht8* allele, and our PCA analysis revealed that there is a relatively large genetic distance between them and the bulk of the GrII varieties (Figure 4). Using these varieties as crossing partners for *Rht8* introduction into other high yielding Northwestern European varieties could thus be a way to add genetic diversity and novel dwarfing alleles without resorting to varieties bred for very different climates.

## Supporting Information

Figure S1 Genetic diversity structure of the 92 hexaploid wheat genotypes. Based on the output of a Bayesian algorithm implemented in the program STRUCTURE using the reduced set of 695 markers. Population memberships for each genotype is shown based on K being two. Bars indicate relation to GrI. Vertical line represents separation between the two groups.
(TIFF)

Figure S2 Linkage disequilibrium (r^2^-values) versus chromosome position for adjacent marker-pairs for the total population. The LD between pairs of adjacent loci is plotted at the locus nearest to zero.
(TIFF)

Figure S3 Linkage disequilibrium (r^2^-values) versus chromosome position for adjacent marker-pairs for GrI. The LD between pairs of adjacent loci is plotted at the locus nearest to zero. Shown for GrI found in population structure analysis.
(TIFF)

Figure S4 Linkage disequilibrium (r^2^-values) versus chromosome position for adjacent marker-pairs for GrII. The LD between pairs of adjacent loci is plotted at the locus nearest to zero. Shown for GrII found in population structure analysis.
(TIFF)

Table S1 Number and size (cM) of gaps among all mapped markers in the wheat DArT array version 3 (Triticarte Pty Ltd).
(DOCX)

Table S2 Distribution of 1,435 polymorphic and mapped DArT markers across the A, B and D genome.
(DOCX)

Table S3 Private alleles detected in the varieties among the 1,849 polymorphic DArT markers.
(DOCX)

Table S4 Analysis of molecular variance (AMOVA) for structure groups p = 0.001.
(DOCX)

Table S5 Significant DArT markers associated with population structure with two subgroups.
(DOCX)

Table S6 Xgwm261(*Rht8*) genotype.
(DOCX)

Dataset S1 Excel file showing the raw genotyping results.
(XLSX)
