# Supplementary figures and images for: Genetic Diversity and Population Structure Analysis of European Hexaploid Bread Wheat (Triticum aestivum L.) Varieties
Source: PLoS One. 2014 Apr 9;9(4):e94000. doi: 10.1371/journal.pone.0094000 (PMC3981729; doi:10.1371/journal.pone.0094000)

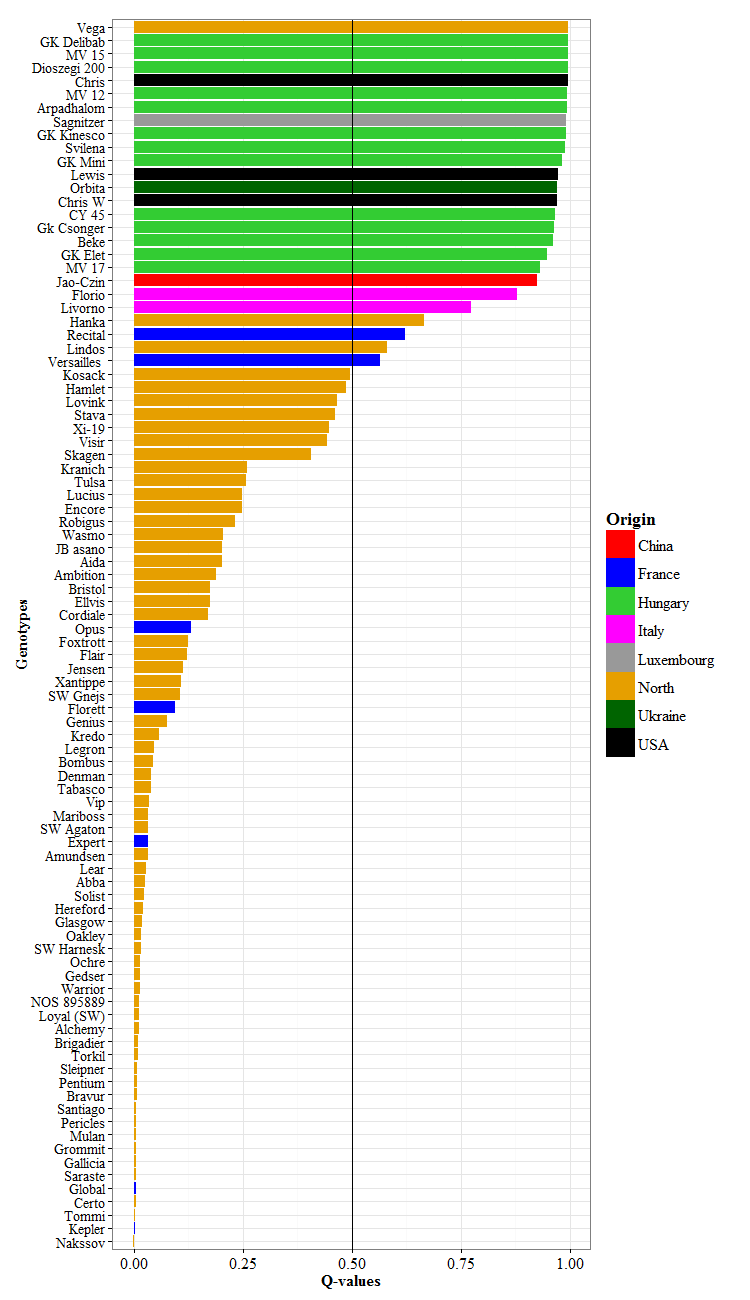

Supplement: Figure S1 — Genetic diversity structure of the 92 hexaploid wheat genotypes. Based on the output of a Bayesian algorithm implemented in the program STRUCTURE using the reduced set of 695 markers. Population memberships for each genotype is shown based on K being two. Bars indicate relation to GrI. Vertical line represents separation between the two groups. (TIFF) [file pone.0094000.s001.tiff]

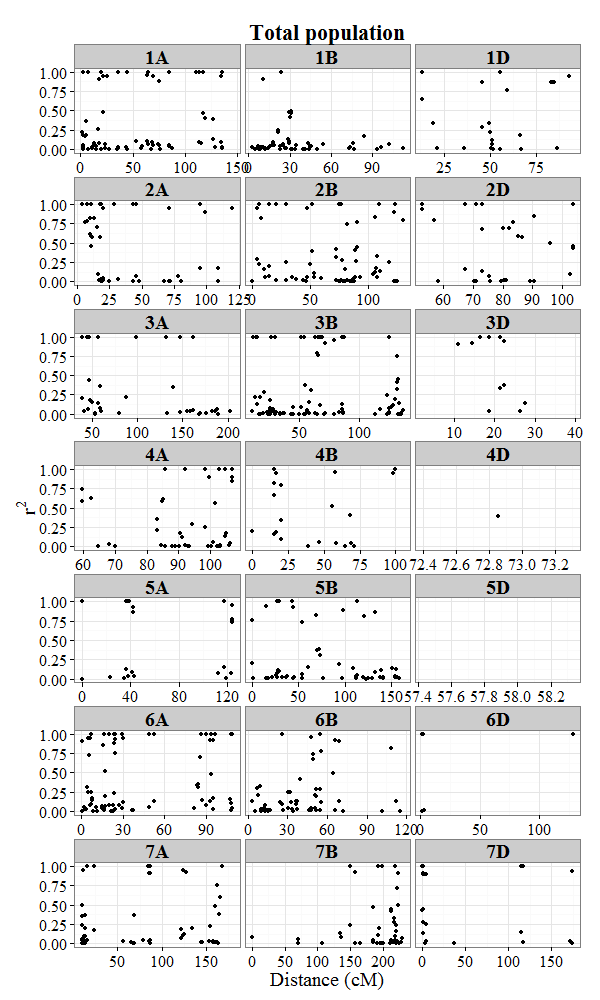

Supplement: Figure S2 — Linkage disequilibrium (r2-values) versus chromosome position for adjacent marker-pairs for the total population. The LD between pairs of adjacent loci is plotted at the locus nearest to zero. (TIFF) [file pone.0094000.s002.tiff]

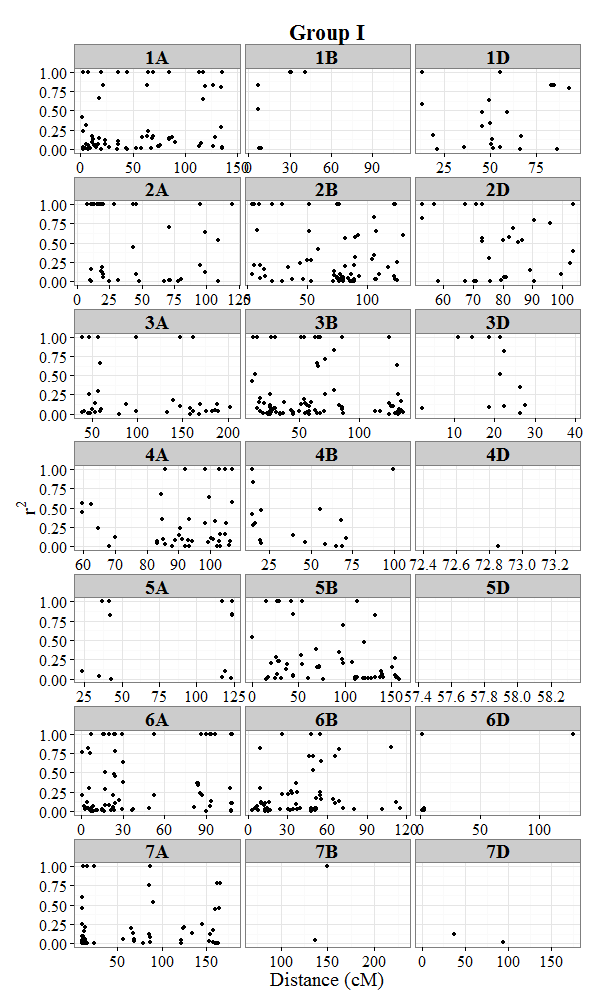

Supplement: Figure S3 — Linkage disequilibrium (r2-values) versus chromosome position for adjacent marker-pairs for GrI. The LD between pairs of adjacent loci is plotted at the locus nearest to zero. Shown for GrI found in population structure analysis. (TIFF) [file pone.0094000.s003.tiff]

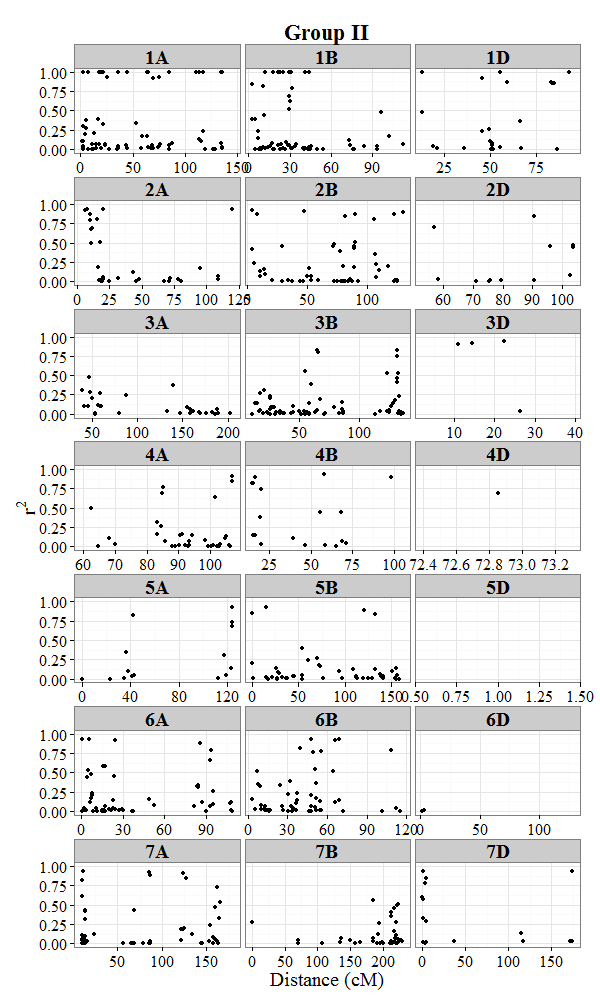

Supplement: Figure S4 — Linkage disequilibrium (r2-values) versus chromosome position for adjacent marker-pairs for GrII. The LD between pairs of adjacent loci is plotted at the locus nearest to zero. Shown for GrII found in population structure analysis. (TIFF) [file pone.0094000.s004.tiff]
