# Supplementary material for: Genetic Diversity and Population Structure Analysis of European Hexaploid Bread Wheat (Triticum aestivum L.) Varieties
Source: PLoS One. 2014 Apr 9;9(4):e94000. doi: 10.1371/journal.pone.0094000 (PMC3981729; doi:10.1371/journal.pone.0094000)
Supplement: Table S1 — Number and size (cM) of gaps among all mapped markers in the wheat DArT array version 3 (Triticarte Pty Ltd). (DOCX) [file pone.0094000.s005.docx]

**Table S1.** Number and size (cM) of gaps among all mapped markers in the wheat DArT array version 3 (Triticarte Pty Ltd)

|  |  | **A** | **B** | **D** | **Whole genome** |
| --- | --- | --- | --- | --- | --- |
| 0-10 |  | 1512 | 2193 | 826 | 4531 |
| 10-20 |  | 9 | 1 | 13 | 23 |
| 20-30 |  | 3 | 0 | 7 | 10 |
| 30-40 |  | 0 | 0 | 4 | 4 |
| 40-50 |  | 1 | 0 | 0 | 1 |
| >50 |  | 0 | 0 | 1 | 1 |
| **Total** |  | **1525** | **2194** | **851** | **4570** |
