# Supplementary material for: Genetic Diversity and Population Structure Analysis of European Hexaploid Bread Wheat (Triticum aestivum L.) Varieties
Source: PLoS One. 2014 Apr 9;9(4):e94000. doi: 10.1371/journal.pone.0094000 (PMC3981729; doi:10.1371/journal.pone.0094000)
Supplement: Table S2 — Distribution of 1,435 polymorphic and mapped DArT markers across the A, B and D genome. (DOCX) [file pone.0094000.s006.docx]

**Table S2.** Distribution of 1,435 polymorphic and mapped DArT markers across the A, B and D genome.

|  | **Number** | **%** | **Total distance**  **(cM)** | **Mean marker distance**  **(cM/marker)** |
| --- | --- | --- | --- | --- |
| A | 602 | 42 | 876 | 1.5 |
| B | 617 | 43 | 977 | 1.6 |
| D | 215 | 15 | 670 | 3.1 |
| **Total** | 1435 | 100 | 2523 | 1.8 |
