# Supplementary material for: Genetic Diversity and Population Structure Analysis of European Hexaploid Bread Wheat (Triticum aestivum L.) Varieties
Source: PLoS One. 2014 Apr 9;9(4):e94000. doi: 10.1371/journal.pone.0094000 (PMC3981729; doi:10.1371/journal.pone.0094000)
Supplement: Table S3 — Private alleles detected in the varieties among the 1,849 polymorphic DArT markers. (DOCX) [file pone.0094000.s007.docx]

**Table S3.** Private alleles detected in the varieties among the 1,849 polymorphic DArT markers.

| **Accession** | **Chromosome** | **Marker ID** |
| --- | --- | --- |
| Ochre | 1A | wPt-732113 |
|  |  | wPt-734090 |
| Kranich | 6A | wPt-729773 |
|  | 3D | wPt-741698 |
|  | Unknown | wPt-731553 |
| Florio | 3A | wPt-799851 |
|  | 2B | tPt-9948 |
|  | 7A | wPt-1119 |
|  |  |  |
