# Supplementary material for: Genetic Diversity and Population Structure Analysis of European Hexaploid Bread Wheat (Triticum aestivum L.) Varieties
Source: PLoS One. 2014 Apr 9;9(4):e94000. doi: 10.1371/journal.pone.0094000 (PMC3981729; doi:10.1371/journal.pone.0094000)
Supplement: Table S4 — Analysis of molecular variance (AMOVA) for structure groups p = 0.001. (DOCX) [file pone.0094000.s008.docx]

**Table S4.** Analysis of molecular variance (AMOVA) for structure groups p= 0.001

|  | **df** | **SS** | **MS** | **Est. Var.** | **%** |
| --- | --- | --- | --- | --- | --- |
| **Among Pops** | 1 | 971 | 971 | 22 | 14% |
| **Within Pops** | 90 | 12275 | 136 | 136 | 86% |
| **Total** | 91 | 13245 |  | 159 | 100% |
