# Supplementary material for: Genetic Diversity and Population Structure Analysis of European Hexaploid Bread Wheat (Triticum aestivum L.) Varieties
Source: PLoS One. 2014 Apr 9;9(4):e94000. doi: 10.1371/journal.pone.0094000 (PMC3981729; doi:10.1371/journal.pone.0094000)
Supplement: Table S5 — Significant DArT markers associated with population structure with two subgroups. (DOCX) [file pone.0094000.s009.docx]

**Table S5.** Significant DArT markers associated with population structure with two subgroups.

| **DArT marker from our results** | **Chrom** | **Pos** | **Link with marker from Crossa et al.** | **Trait from Crossa et al.** |
| --- | --- | --- | --- | --- |
| wPt-5577 | 1A | 129 | 3 cM from wPt-8016 | LR and *Pm17* |
| wPt-1912* | 1B | 7 | 2 cM from wPt-7094 | 1B/1R, Glu-3 |
| wPt-3855 | 1D | 21 | No link found |  |
| wPt-741323, wPt-731267 | 1D | 50 | 4 cM from wPt-9380 | GY, *Yr25* |
| wPt-6687 | 2A | 109 | Same marker | GY, YR (*Yr17*, *Yr32* and *Ppd3*) |
| wPt-8460 | 2B | 77 | Between wPt-3986 and wPt-4125, Markers in between relates to GY | GY |
| wP-8393, wPt-6850, wPt-733674,  wPt-5586, wPt-733932, wPt-2781,  wPt-6847, wPt-668120, wPt-664745,  wPt-665317, wPt-668044, wPt-731130 | 2D | 67-89 | These two markers are flanking wPt-4144 | GY, *Ppd1*/*Rht8* |
| wPt-8892 | 3A | 186 | 11 cM from wPt-4407 | GY |
| wPt-3195 | 3A | 202 | Around wPt-1688 | *Eps* |
| wPt-3810 | 4A | 60 | No link found |  |
| wPt-2903wPt-669203, wPt-7327 | 4A | 83-86 | Several of our markers flank region with Wpt-2084 | GY, *Yr37* |
| wPt-4424 | 4A | 98 | Around wPt-5172 | PM |
| wPt-1912* | 4B | 20 | 4 cM away from wPt-1272 | LR, *Lr12*, *Lr31*, *Rht1* |
| wPt-2607, wPt-3457 | 5B | 70;73 | Around wPt-7101 | GY |
| tPt-1253 | 5B | 120 | 8 cm from wPt-9598 | *Vrn3*, *Vrn4*, *Lr18* |
| wPt-8719, wPt-731934 | 6A | 16;20 | 3 cM from wPt-7623 | *Yr38*, *Sr13*, *Sr26* |
| wPt-729806 | 6A | 37 | No link found |  |
| wPt-5480 | 6A | 90 | 3 cM from wPt-1642 | GY, *Yr38* |
| wPt-7489 | 6B | 55 | No link found |  |
| wPt-7785 | 7A | 57 | No link found |  |
| wPt-5524 | 7A | 134 | No link found |  |

DArT marker map from our study were linked to DArT-marker map by Crossa et al. [46]. Markers in Crossa et al. [46] are linked to agronomic genes.

Marker association is shown for yellow rust (YR), Leaf rust (LR), grain yield (GY), stripe rust (SR), Plant height (PH), powdery mildew (PM), vernalization genes (*VrA4, VrA4*), 1B/1R wheat-rye translocations (1B/1R), glutenin encoding genes (*Glu-3*), photoperiod genes (*Ppd1*), reduced height genes (*Rht1* and *Rht8*). Earliness *per se* (*Eps*)

* Found in GLM association between markers and subgoups.
