# Supplementary material for: Genetic Diversity and Population Structure Analysis of European Hexaploid Bread Wheat (Triticum aestivum L.) Varieties
Source: PLoS One. 2014 Apr 9;9(4):e94000. doi: 10.1371/journal.pone.0094000 (PMC3981729; doi:10.1371/journal.pone.0094000)
Supplement: Table S6 — Xgwm261(Rht8) genotype. (DOCX) [file pone.0094000.s010.docx]

**Table S6.** Xgwm261 (*Rht8*) genotype.

| **162** | **174** | **192** |
| --- | --- | --- |
| 1,3,5,8,9,11,14,17,18,19,23,24,  25,26,28,31,42,44,46,50,56,58,  60,66,68,69,75,91,93 | 6,7,12,13,15,15,20,21,22,27,29,32,33,34,  35,36,37,38,39,40,43,45,47,48,49,51,53,  54,55,57,59,73,74,76,77,78,79,81,83,84,  85,86,87,88,89,90,92 | 2,4,30,41,52,61,62,63,64,65,  67,70,71,72,80,82 |

Numbers refer to accessions in Table 1. Missing accessions did not amplify.
